# Role of CT in the Staging of Colorectal Tumors: A Preliminary Study on 10 Dogs

**DOI:** 10.3390/ani14111521

**Published:** 2024-05-21

**Authors:** Simone Perfetti, Martina Mugnai, Simonetta Citi, Laura Marconato, Armando Foglia, Silvia Sabattini, Nikolina Linta, Alessia Diana

**Affiliations:** 1 Department of Veterinary Clinical Science, University of Bologna, 40064 Ozzano dell’Emilia, Italy; laura.marconato@unibo.it (L.M.); armando.foglia2@unibo.it (A.F.); silvia.sabattini@unibo.it (S.S.); nikolina.linta2@unibo.it (N.L.); alessia.diana@unibo.it (A.D.); 2 AniCura Istituto Veterinario di Novara, 28060 Granozzo con Monticello, Italy; martina.mugnai@anicura.it; 3 Department of Veterinary Clinical Science, University of Pisa, 56100 Pisa, Italy; simonetta.citi@unipi.it

**Keywords:** computed tomography, colorectal, oncology

## Abstract

**Simple Summary:**

Colorectal tumors are rare in dogs and are difficult to diagnose early due to their non-specific symptoms. There are limited studies on the CT features of these tumors in veterinary medicine. This study aims to describe the CT features of colorectal tumors in dogs and evaluate CT’s usefulness in tumor staging. This study shows how CT helps identify colorectal lesions, assess lymph node involvement, and determine if other organs are affected. It also describes the common CT features of different types of colorectal tumors, aiding oncologists in diagnosis and staging, and surgeons in planning surgeries more effectively.

**Abstract:**

This study aimed to define the CT features of colorectal tumors in dogs and assess CT’s role in tumor staging. It was a retrospective, multicenter, descriptive study involving dogs with a cyto-histopathological diagnosis of colorectal tumors and high-quality pre- and post-contrast CT scans of the abdomen. CT successfully identified colorectal lesions in all cases, showing variations such as wall thickening, presence of masses, and luminal stenosis. It also detected lymph node involvement. Overall, this study helps us to understand the CT features of both epithelial and mesenchymal colorectal tumors, emphasizing CT’s importance in staging and surgical planning for affected dogs. Larger studies are needed to identify specific CT findings for different colorectal neoplasms.

## 1. Introduction

Colorectal tumors are rare in dogs, accounting for less than 2% of all canine tumors. Early diagnosis of colorectal tumors in dogs presents a significant challenge due to their nonspecific clinical presentation, which includes weight loss, diarrhea, anorexia, hematochezia, and tenesmus [1]. Adenocarcinoma is the most prevalent histotype, while leiomyosarcoma is the most common mesenchymal gastrointestinal tumor in dogs [2,3].

Traditionally, colonoscopy has been deemed a valuable preoperative diagnostic tool for assessing dogs with colorectal masses. It helps characterize the location and nature of the disease, assess the presence of multiple lesions, and obtain biopsy samples to inform clinical decision making. The selection of a surgical approach in dogs is usually determined by the location and extent of the lesion, often evaluated through direct colonoscopic visualization [4]. However, relying solely on colonoscopic localization can be inaccurate, especially in terms of distance measurements. Additionally, it does not provide information on loco-regional or distant spread, hindering accurate staging. Furthermore, in both humans and veterinary oncology patients, staging using computed tomography (CT) examination is preferred over other methods because it offers more a precise investigation of the primary tumor, lymph nodes, and other metastatic organs [5,6,7].

In human medicine, CT is an effective technique for determining the precise localization and extension of colorectal carcinoma (CRC), enabling the evaluation of metastases, and facilitating a fairly accurate staging of the neoplasm [8]. In veterinary medicine, the use of CT for investigating the gastrointestinal tract may be more sensitive and specific than other conventional diagnostic methods, such as ultrasound, which is hindered by interference from surrounding bony structures within the pelvic canal, and standard radiographs alone, which lack the precision to differentiate soft tissue structures [5,9,10].

A recent study describing the CT characteristics of spindle, epithelial, and round cell tumors of the canine gastrointestinal tract revealed overlapping findings among different tumor types [11]. Contrast-enhanced CT provided valuable information for characterizing tumors based on their site of origin, extent of local invasion, and presence of distant metastases [11]. To our knowledge, there are no studies specifically investigating CT findings of colorectal tumors in dogs. Therefore, the objective of this retrospective study is to define the computed tomographic features of colorectal tumors in dogs and assess the utility of CT in tumor staging.

## 2. Materials and Methods

### 2.1. Population

In this retrospective, multicenter, descriptive study, dogs included were those referred to the Veterinary Teaching Hospital (VTH) of the University of Bologna, University of Pisa, and AniCura Istituto Veterinario di Novara between February 2018 and January 2024. An electronic database search was conducted for dogs across all the institutions that underwent CT examination of the abdomen for colorectal neoplasia. The inclusion criteria for dogs included having a cyto-histopathological diagnosis of colorectal tumor and the availability of high-quality pre- and post-contrast CT examinations of the abdomen for review. For each dog, data regarding signalment, clinical history, and surgical (if available) findings were recorded.

### 2.2. CT Protocol

The CT examinations were conducted under general anesthesia with dogs placed in sternal recumbency. The animals were fasted for approximately 12 h before anesthesia. Dogs were pre-anesthetized with an intramuscular administration of dexmedetomidine (2 mcg/kg) alone or in combination with methadone (0.2 mg/kg). They were induced using propofol (2.0 mg/kg) administered intravenously (IV) and intubated with a tracheal tube. General anesthesia was maintained using isoflurane (1.5%: vaporizer setting) and oxygen (2 L/min) under mechanical ventilation.

The exams were performed using three different CT scanners (Philips Diamond Select Brilliance 64, Amsterdam, The Netherlands; 16-slice Revolution Act, GE Healthcare, Medical System, Milan, Italy; and Optima 660, GE Healthcare, Medical System, Milan, Italy). When clinically indicated, pneumocolonography was performed, following the methodology previously described in the literature [12,13]. The acquisition parameters were set as follows: helical mode, 120–140 kVp, exposure 200–250 mAs, 1.5 mm slice thickness, 0.75 mm spacing, 512 × 512 matrix, and a medium filter algorithm. Contrast injection was carried out after intravenous administration of a nonionic iodinated contrast agent (Iopamiro 300 mg/mL) through a cephalic vein catheter, employing either a manual injection or a dual-contrast power injector. Each dog received a contrast agent at a dose of 2 mL/kg body weight, corresponding to 600 mg Iodine/kg body weight. In cases where a manual injection was performed, the scan delay was set to 40 s after the start of contrast agent administration. Alternatively, when a power injector and a bolus tracking technique were employed, with a flow rate of 2–2.5 mL/s, a region of interest (ROI) was placed within the descending aorta, and the scan commenced when a mean attenuation of 100 HU was reached.

### 2.3. Data Analysis

CT images were transferred to a workstation using a commercially available DICOM imaging viewing software (Osirix MD v 9.0.1, Pixmeo SARL, 266 Rue de Bernex, CH1233 Bernex, Switzerland), and were analyzed by authors SP and AD. Each study was evaluated with a soft tissue window (Window level: +50; Window width: 400). For each case, the appearance of the colorectal lesion, lymph nodes, surrounding organs, and peritoneum were assessed. Findings related to the colorectal lesion were recorded, including location, extension (diffuse if the length exceeded 40 cm; segmental if the length was between 5 and 40 cm; or focal if the length was less than 5 cm), feature (transmural [circumferential, symmetric, or asymmetric]; endophytic, or exophytic), maximum thickness, attenuation (presence or absence of mineralized foci), contrast medium enhancement, presence or absence of luminal stenosis and adherences or invasion of surrounding structures. Regarding regional lymph nodes, the evaluation included: identification of involved lymph nodes, assessment of shape and axial diameters, measurement of attenuation, and observation of contrast medium enhancement. Additionally, the presence or absence of suspicious metastatic involvement in other organs was documented.

## 3. Results

### 3.1. Population

Ten dogs diagnosed with colorectal tumors were included in this study. There were three mixed-breed dogs and one each of the following breeds: Epagnieul Breton, Australian Shepherd, Border Collie, Golden Retriever, Greyhound, Cirneco dell’Etna and Italian Volpino.

Seven dogs were males (of which four were neutered) and three were females (of which two were neutered). The median age was 10 years (range 5–15 years).

The diagnosis was obtained following surgical biopsy in 6 dogs and endoscopic biopsy in 4. There were six adenocarcinomas, two leiomyosarcoma, and one each of squamous cell carcinoma and papillary carcinoma.

Among the six surgically treated dogs, five underwent partial colectomy, and one a pull-out technique. Only in four cases histological (three cases) or cytological (one case) examination of the abdominal lymph nodes was available; only one case showed metastatic lymph nodal involvement (left medial iliac lymph node in a dog with colorectal adenocarcinoma). Therefore, only the presence of lymphadenomegaly was recorded.

### 3.2. Computed Tomographic Findings

**Figure 1:**
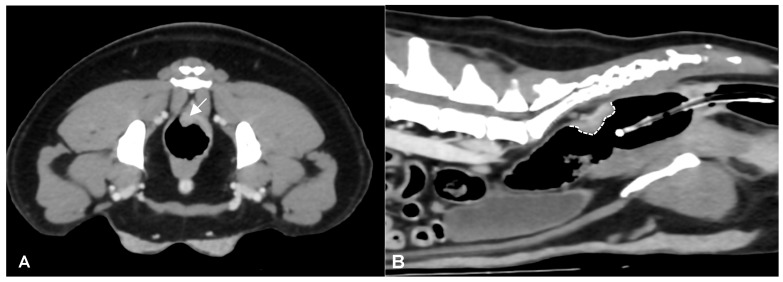
Post-contrast CT images of a colorectal adenocarcinoma in a 5-year-old female Border Collie. Transverse (**A**) and sagittal (**B**) plane reconstructions are available. A transmural, endophytic, broad-based, asymmetric, heterogeneously enhancing thickening of the left dorsolateral aspect of the colorectal wall is noted (white arrow). In the sagittal plane, the craniocaudal extension of the lesion is evident (dotted white line). A Foley catheter is also visible within the gas-filled rectal lumen.

**Figure 2:**
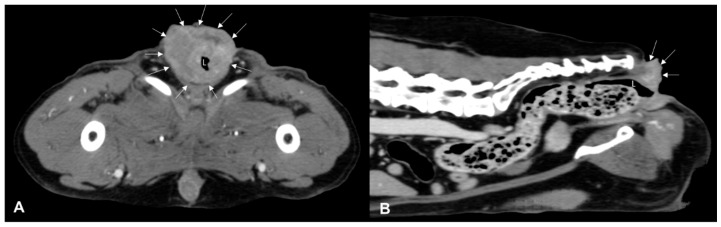
Post-contrast CT images of an anorectal squamous carcinoma in a 15-year-old neutered male Epagneul Breton. Transverse (**A**) and sagittal (**B**) plane reconstructions are available. A transmural, circumferential, asymmetric, heterogeneously enhancing expansile lesion is observed (white arrows). On the sagittal plane, the lesion exhibits a mild exophytic appearance and encircles the anal sac and borders, effacing any adipose cleavage with the tail muscles. L—lumen.

**Figure 3:**
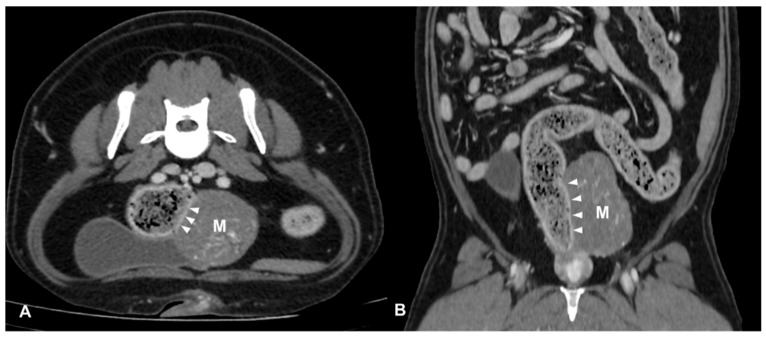
Post-contrast CT images of a colic mesenchymal tumor (leiomyosarcoma) in an 11-year-old mixed breed neutered male dog. Transverse (**A**) and dorsal (**B**) plane reconstructions are available. An expansile, heterogeneously enhancing soft tissue opacity lesion (M) is seen in continuity with the left lateral wall descending colon (arrows heads). The mass is exophytic and involves the outer layer of the colic wall. Multiple mineral foci are observed within the lesion.

CT identified a colorectal lesion in all dogs. Specifically, 5 dogs had lesions localized at the colorectal junction, 3 at the descending colon, 1 at the transverse colon, and 1 at the anorectal junction. The characteristics of these lesions varied: 6 (Figure 1) dogs exhibited transmural, circumferential, asymmetric thickening of the wall, while 2 (Figure 2) cases (one adenocarcinoma and one carcinoma) displayed transmural, circumferential symmetric thickening. The leiomyosarcoma case was characterized by the presence of an exophytic mass (Figure 3).

The thickening of the lesions ranged from 7 to 49 mm with a median thickness of 19 mm, and the extension ranged from 23 to 84 mm with a median extension of 50 mm. Concerning extension, 5 lesions were classified as focal, while the other 5 were classified as segmental. Nine lesions resulted in the loss of visualization of the normal wall stratigraphy, while only one case of leiomyosarcoma retained recognizable wall layers.

The attenuation value of the lesion ranged from 40 to 57 HU with a median attenuation value of 46.5 HU, with heterogeneous attenuation in 7 (70%) dogs and homogeneous attenuation in 3 (30%). In two (20%) dogs, intralesional mineralization foci were observed. All lesions showed heterogeneous enhancement post-contrast medium administration, with attenuation values ranging from 70 to 105 HU and a median attenuation value of 97.5 HU. Tortuous vessels were observed intralesionally or peripherally in all three cases for which the arterial phase was available.

Luminal stenosis was noted in all cases except for two: the descending colic leiomyosarcoma and the colorectal adenocarcinoma. In one case of anorectal squamous cell carcinoma, the infiltration of surrounding structures such as anal sacs and tail muscles was observed. Additionally, adherences between the lesion and surrounding structures like the urinary bladder, sacro-caudal muscles and epaxial muscles, tail of the spleen, and/or left external iliac artery were noted in 4, 3, and 1 dog, respectively.

Tributary lymph nodes involvement was noted in nine dogs, with the caudal mesenteric and sacral lymph nodes being the most commonly affected. Additionally, colic and jejunal lymphadenopathy were observed in 3 dogs, while medial iliac lymph node involvement was suspected in 2 cases. All lymph nodes were enlarged, with thickness ranging from 5 to 13 mm for the caudal mesenteric lymph node, 6 to 11 mm for colic lymph nodes, 10 to 13 mm for the sacral lymph node, 8 to 14 mm for jejunal lymph nodes, and 9 to 10 mm for medial iliac lymph nodes. Jejunal and medial iliac lymph nodes were characterized by elongated shape, while colic, caudal mesenteric, and sacral lymph nodes exhibited a globular shape. In eight (80%) dogs, lymph nodes showed homogeneous enhancement in the pre-contrast series, except for one dog with colorectal adenocarcinoma, where jejunal, colic, and caudal mesenteric lymph nodes exhibited heterogeneity. After contrast medium administration, lymph nodes in four dogs exhibited heterogeneous enhancement, while in four cases, the enhancement was homogeneous. In one case, the enhancement was heterogeneous for jejunal lymph nodes and homogeneous for the caudal mesenteric lymph node. No involvement of other abdominal organs was documented in all cases, except for one case where two areas compatible with steatonecrosis were observed near the ileocolic junction.

### 3.3. Surgical Procedures and Findings

Six dogs underwent surgery for the removal of the colorectal lesion. In five dogs with colic or colorectal lesions, a celiotomic or xipho-pubic surgical access was performed in relation to location of the lesion, with dogs placed on dorsal recumbency. In these cases, a partial colectomy and a colo-colonic or colo-rectal anastomosis was performed. In a single dog with an anorectal tumor, a pull-out technique was performed. From surgical reports available for review (four cases), the surgical findings were consistent with CT findings in all dogs especially regarding the location, size, and extension of the lesion. The suspected adherences detected on CT were also confirmed at surgical examination.

## 4. Discussion

The aim of this study was to characterize the CT findings of colorectal tumors in dogs, contributing to the understanding and staging of these tumors. Our study identified colorectal adenocarcinoma as the most prevalent tumor, consistent with the existing literature [3]. In our population, dogs affected by colorectal tumors had a mean age of 9.6 ± 3 years, aligning with the literature’s reports that suggest a mean age of 8 years for dogs with similar conditions [3,14].

The employed CT protocols proved a comprehensive assessment of the lesions in all cases.

The use of CT pneumocolonography, as documented in the literature, proved advantageous in achieving better distension of the wall and improved visualization of lesion margins [10,12,13]. However, in all cases in which CT pneumocolonography was performed, the wall exhibited less distensibility at the site of the wall lesion compared to other colorectal segments. This technique, already performed by Simeoni et al., in one dog affected by a histologically confirmed adenocarcinoma of the descendent colon, showed good results in terms of mass visualization and lumen distension [10].

In all cases, both pre- and post-contrast CT scans were conducted. Nonetheless, a bolus tracking technique for contrast medium administration was employed only in two cases. This technique enhanced the visualization of intralesional vascularization and mucosal enhancement of the gastrointestinal tract, facilitating a more precise evaluation of the lesion’s margins. In the only two cases with an available arterial phase, the presence of small intralesional or peripheral tortuous vessels was observed, indicative of intense neoangiogenesis. In human medicine, both magnetic resonance imaging (MRI) and CT are recognized as valuable modalities for evaluating tumoral neoangiogenesis, aiding in cancer diagnosis, staging, and monitoring treatment response [15]. In our study, adenocarcinoma predominantly occurred at the colorectal junction, aligning with the literature where this location is commonly reported [1,3,16,17]. Only one adenocarcinoma was located in the middle third of the descending colon. The two mesenchymal tumors included in this study were located at the transverse and descending colon, respectively. In dogs, gastrointestinal leiomyosarcomas are typically more common in the stomach and small intestine, while GISTs are more commonly located in the caecum [16]. All cases, except one, exhibited a single lesion characterized by a complex aspect with circumferential transmural predominantly asymmetric thickening. The exception was a case of colorectal leiomyosarcoma characterized by a voluminous exophytic mass. Notably, despite the observation of a single lesion in all cases, previous studies in dogs with colorectal epithelial tumors reported that 12% to 19% had multiple colorectal masses [17,18]. This could be due to the small sample size, which does not represent the whole canine population, or due to possible early diagnosis of the colorectal tumor in our population.

Mean pre- and post-contrast attenuation values were consistent across all lesions, with mean values of 48 HU and 92 HU, respectively. While there are no specific studies in veterinary medicine for comparison, these values are quite similar to those reported for gastric adenocarcinoma in dogs [19]. In the current study, all lesions exhibited heterogeneous enhancement, consistent with the findings reported in a recent study, particularly in relation to epithelial tumors [11].

Intralesional mineralization foci were present in two cases (squamous cell carcinoma and leiomyosarcoma), and luminal stenosis was observed in all cases except for the dog with an exophytic leiomyosarcoma. Luminal stenosis is a common finding in other studies of gastrointestinal tumors in both epithelial and mesenchymal neoplasia [13,18,20].

In all cases, lymph node involvement was observed, with the caudal mesenteric, colic, jejunal, sacral, and medial iliac lymph nodes being most frequently affected. In human medicine, irregularly margined and enlarged lymph nodes are suggestive of metastatic spread, and the presence of a cluster of three or more lymph nodes with irregular margins without lymphadenomegaly is considered pathological [6]. Distant metastases were not observed in any case. While CT is useful for evaluating the colorectal lesion and detecting locoregional and distant metastases, lymph node involvement remains challenging to assess definitively using only CT findings. In human medicine, histopathological evaluation is considered the gold standard for lymph node involvement, as highlighted by studies indicating the limitations of CT in accurately determining the extent of lymph node metastasis [6].

This study has some limitations, mainly due to its multicentric and retrospective nature. In particular, the lack of standardized CT protocols prevented us from obtaining detailed vascular information for each colorectal lesion, such as the absence of a bolus tracking technique. Additionally, the absence of histological and/or cytological examination of the abdominal lymph nodes hindered the correlation between CT and cyto-histological findings. The limited number of cases, particularly in the mesenchymal tumors group, precluded statistical analysis.

## 5. Conclusions

In conclusion, this descriptive study contributes to delineating the CT characteristics of both epithelial and mesenchymal colorectal tumors, demonstrating the utility of CT in staging and surgical planning for dogs with such tumors. Further studies with a larger sample size are needed to identify specific findings for certain colorectal neoplasms.

## Data Availability

The data are included in this study. CT images can be requested from simone.perfetti4@unibo.it.
